# Development of a FRET-Based Assay for Human Neutral Sphingomyelinase 2

**DOI:** 10.1021/acs.biochem.6c00322

**Published:** 2026-08-07

**Authors:** Khalayi Martha Aywa, Christian Kappe, Botheina Ghandour, Shujuan Gao, Christoph Arenz, Yusuf A. Hannun, Michael V. Airola

**Affiliations:** † Department of Biochemistry and Cell Biology, 12301 Stony Brook University, Stony Brook New York 11794, United States; ‡ Stony Brook University Cancer Center, Stony Brook, New York 11794, United States; § Institute of Chemistry, 9373 Humboldt-Universität zu Berlin, Brook-Taylor-Strasse 2, 12489 Berlin, Germany

## Abstract

Neutral sphingomyelinase 2 (nSMase2) is a membrane-bound enzyme that hydrolyzes sphingomyelin (SM) into ceramide and phosphocholine. By generating the bioactive lipid ceramide, nSMase2 plays a critical role in cell stress responses and in regulating exosomes that package and transfer pathogenic factors, including tau protein and amyloid β. Thus, nSMase2 has been implicated in Alzheimer’s disease and other neurological disorders. However, current tools to measure nSMase2 activity are limited, in particular those that could be used in therapeutic development. Here we developed a high-throughput assay to measure human nSMase2 activity. The assay uses a sphingomyelin substrate analogue with a FRET donor and acceptor pair attached to the headgroup and acyl-chain, respectively. Using recombinant human nSMase2, we sensitively detected both wild-type and mutant activity and demonstrated inhibition by the known nSMase2 inhibitor GW4869. The assay captures the major hallmarks of nSMase2 regulation by anionic lipids and displays sensitivity capable of detecting nSMase2 activity from cell lysates. Together, this assay enables rapid screening of nSMase2 inhibitors to support future therapeutic development.

## Graphical abstract

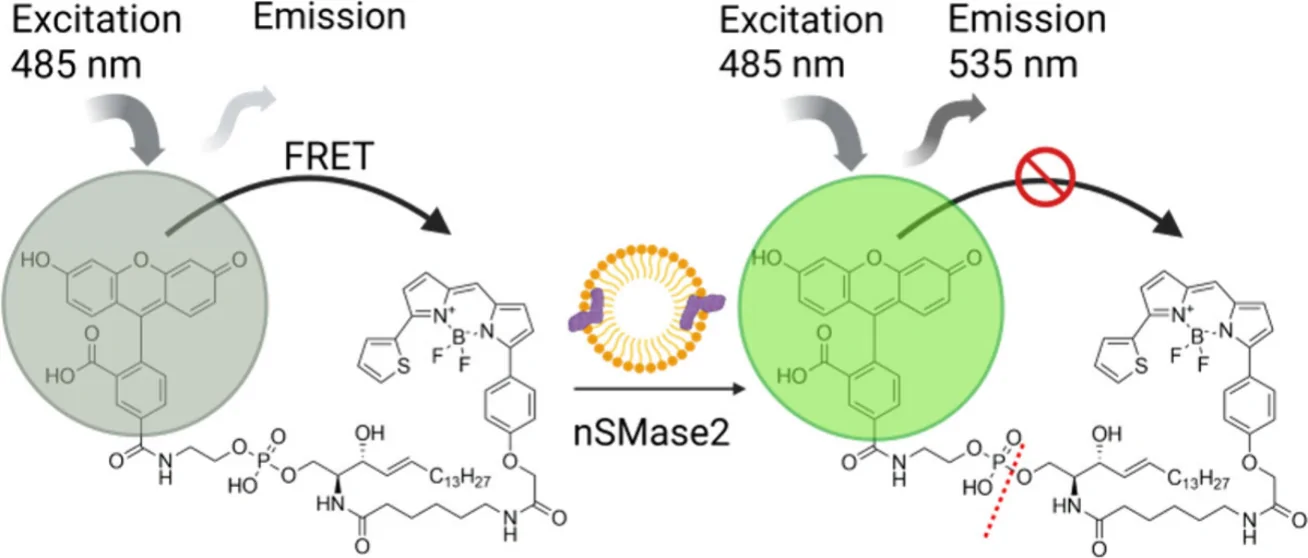

## Introduction

Ceramide is a bioactive lipid mediator involved in various cellular processes including cellular stress response.^1,2^ A major source of ceramide is the hydrolysis of sphingomyelin (SM) into ceramide and phosphocholine by sphingomyelinases (SMases), which are broadly categorized according to their pH optima for activity into acid, neutral and alkaline.^3–6^ Under stress conditions, such as oxidative stress, hypoxia, inflammation, and chemotherapy,^7–12^ ceramide is rapidly generated mainly by neutral sphingomyelinase 2 (nSMase2, *SMPD3* gene). nSMase2 is highly expressed in the brain^13^ and other tissues.^14^ In the cell, nSMase2 localizes to the plasma membrane^10,15–17^ and Golgi apparatus.^13,18^

nSMase2 is involved in multiple processes, including cell differentiation,^8^ epithelial to mesenchymal transition,^19^ and the biogenesis of extracellular vesicles,^20,21^ some of which contain pathogenic cargo such as tau protein^22^ and β-amyloid.^23^ Accordingly, nSMase2 has been linked to cancer,^24^ cardiovascular,^25^ and neurological pathologies^26^ including Alzheimer’s disease, Parkinson’s disease, and cerebral ischemia.^27^ nSMase2 has been implicated in Alzheimer’s disease (AD) pathogenesis through several mechanisms, including extracellular vesicle biogenesis,^20,28^ amyloid β production,^23,29–31^ tau seeding,^22^ and neuroinflammation.^32^ Lipidomic analyses have revealed decreased sphingomyelin (SM) and increased ceramide levels in the sera and brains of individuals with AD.^26,33,34^ Since nSMase2 contributes to multiple mechanisms implicated in AD,^35^ it is a promising therapeutic target.

Biochemically, nSMase2 has been well characterized, with requirements for Mg^2+^ as a cofactor and activation by specific lipids.^13^ Human nSMase2 is a monotopic enzyme with a soluble catalytic domain and a membrane-embedded N-terminal hydrophobic domain (NTD) that embeds within the membrane bilayer but does not traverse it.^17,36^ The NTD contains two hydrophobic segments and a juxtamembrane region, and functions as a membrane anchor, lipid binding domain, and allosteric activator of the catalytic domain.^17^ The NTD binds phosphatidylserine (PS), which is necessary for nSMase2 to be fully active.^13,17,37,38^ Additional studies have demonstrated that the NTD contains motifs that bind the anionic phospholipids PS and phosphatidic acid (PA), which can also stimulate nSMase2 activity.^38^ The most widely used inhibitor of nSMase2 is GW4869, which is a noncompetitive inhibitor of the substrate SM, but a competitive inhibitor with the allosteric lipid activator PS.^39^ GW4869 is a potent inhibitor of nSMase2 but suffers from poor solubility.^40,41^

Current activity assays for nSMase2 utilize radioactively labeled SM as a substrate,^17,42^ fluorescently labeled 7-nitro-2,1,3-benzoxadiazol-4-yl (NBD) SM^43,44^ or 4,4-difluoro-5,7-dimethyl-4-bora-3a,4a-diaza-S-indacene (BODIPY FL) SM,^45^ or invoke a fluorometric coupled enzyme assay (AmplexRed, Cayman, USA) whose final readout is fluorescent resorufin.^46,47^ Existing direct readouts of nSMase2 enzymatic activity have limited ability to monitor enzyme activity continuously in real-time, which has limited detailed kinetic and mechanistic studies. Since coupled assays utilize multiple enzymes that may become rate-limiting and introduce assay-dependent artifacts, this makes them limiting for kinetic studies and screening. Thus, secondary counterscreening is required to increase confidence in hits. The radioactive SM, NBD and BOPIDY labeled SM methods are nonhomogenous and require extraction by a modified Bligh–Dyer organic extraction using chloroform and methanol.^48^ Because these methods require organic extraction, they are not compatible with high-throughput screening (HTS). Altogether, this underscores the need for complementary assays suitable for medium- or high-throughput applications to screen large chemical libraries.

In a recent advance, several SM-based Förster resonance energy transfer (FRET) substrates have been developed to monitor acid sphingomyelinase (aSMase) activity in cells and *in vitro*.^49–52^ In these analogues, the SM headgroup and acyl chain are labeled with a matched donor–acceptor fluorescent dye pair.^49–52^ In this distance-dependent mechanism, excitation of the donor leads to nonradiative energy transfer to the nearby acceptor, producing strong acceptor emission while the substrate remains intact.^53^ Upon aSMase-catalyzed cleavage, FRET efficiency drops, leading to increased donor and decreased acceptor fluorescence emission.^49–53^ FRET probes are advantageous because they allow for ratiometric measurements, since the donor-to-acceptor intensity ratio provides a concentration-independent readout of probe hydrolysis and ultimately, improves the reliability of this assay.^49–52,54^

The applicability of FRET probes to other enzymes of SM metabolism remains underexplored. PCK was developed by Kappe et al. (2020) as a FRET probe that enabled sensitive and real-time monitoring of aSMase activity^50^ (Figure 1). 5-carboxyfluorescein (5-FAM) was attached to the choline analogue headgroup as the donor, while BODIPY TR was incorporated into the fatty acid region of SM as the acceptor^50^ (Figure 1).

**Figure 1:**
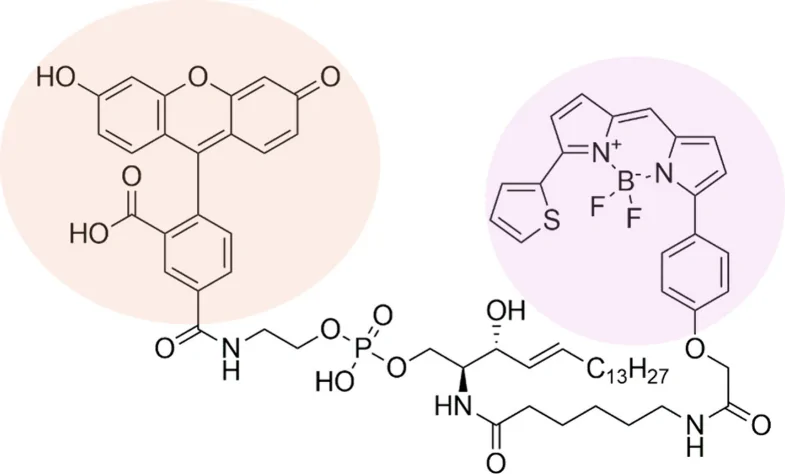
Structure of PCK FRET probe. 5-carboxyfluorescein (5-FAM) is conjugated to the phosphocholine analogue headgroup as the donor fluorophore (brown circle), while BODIPY TR is incorporated into the ceramide fatty acid chain as the acceptor fluorophore (purple circle). Hydrolysis of the phosphodiester bond by nSMase2 separates donor and acceptor, resulting in a decrease in BODIPY TR emission and a corresponding increase in 5-FAM fluorescence emission that is used to monitor enzymatic activity.

To address the limited availability of efficient real-time tools to assess activity of nSMase2, we adapted PCK by modifying assay buffer conditions and developed a FRET-based plate assay suitable for high-throughput inhibitor screening of nSMase2 under the conditions described below. The assay is sensitive and robust, detecting activity from both wildtype nSMase2 and low-activity mutants. Its suitability for inhibitor screening was confirmed using a previously reported nSMase2 inhibitor, GW4869. By adapting an established FRET probe platform to nSMase2, this study not only fills a critical methodological gap but also demonstrates the potential of probes used across different enzymes of the same family to identify distinct enzymatic behaviors. The following results establish the specificity, sensitivity, and mechanistic value of this approach in the context of nSMase2.

## Methods

### PCK Synthesis and Characterization

The PCK FRET probe was synthesized in a multistep procedure as described previously.^50^ Following its final purification by preparative thin layer chromatography, the probe identity was confirmed by HPLC-MS, and ^1^H-, ^19^F- and ^31^P NMR spectra were recorded, all confirming the probe’s chemical structure and purity.

### Plasmid Constructs and Site Directed Mutagenesis

For *E. coli* expression, nSMase2 Δ174-339 was gene synthesized into pET28a vector (BioBasic) using NcoI and XhoI sites, which placed a noncleavable C-terminal 6x His-tag. For mammalian expression, full length human nSMase2 was previously cloned into pEF6 vector as described,^17^ flanked by restriction sites *Bam*HI and Pmel, with a C-terminal V5 tag. The mutants N130A, D430A, R33A Δ45-48, and R92/93A were made by QuikChange site-directed mutagenesis kit (Agilent).

### Protein Expression

BL21-CodonPlus­(DE3)-RIPL cells (Agilent Technologies) were transformed with pET28a vector encoding human nSMase2 1-653 Δ175-339 with a C-terminal 6x His tag. Cultures were grown in Terrific Broth (TB) media supplemented with 0.4% glycerol at 37 °C until an optical density (OD) 600 nm of 1.5 was reached. The temperature was then reduced to 10 °C for 2 h. After 2 h, protein expression was induced with 1 mM Isopropyl-β-D-thiogalactopyranoside (IPTG). Cells were grown for an additional 18 h at 15 °C. To harvest, cells were centrifuged at 3,320 × g for 20 min at 4 °C. The supernatant was decanted, and the cell pellet was stored at −80 °C.

### Protein Purification

Cell pellets were thawed and resuspended in buffer A composed of 10% glycerol (v/v), 50 mM sodium phosphate pH 7.5, 300 mM NaCl, 10 mM β-mercaptoethanol (βME), and 0.5 mM phenylmethylsulfonyl fluoride (PMSF) protease inhibitor. The resuspended cells were sonicated on ice. Cell lysates were centrifuged at 10,000 × g for 30 min at 4 °C to remove cell debris. The supernatant was transferred to a new tube and centrifuged at 105,000 × g for 1 h to isolate membranes. The supernatant was decanted, and the membrane pellet was resuspended in lysis buffer with 1% LMNG and homogenized using a pestle and mortar then incubated overnight with gentle agitation at 4 °C. The next day, the resuspended lysate was then centrifuged at 85,000 × g for 45 min. The supernatant was incubated with nickel-NTA resin with gentle agitation at 4 °C for 1 h. The resin was then transferred to a gravity flow column, and the unbound material was allowed to flow through. The column was subsequently washed with two 10 column volumes (CV) of wash buffer composed of 50 mM sodium phosphate buffer (pH 7.5), 500 mM NaCl, 10 mM βME, 10% glycerol (v/v), 0.02% LMNG, and 20 mM or 40 mM imidazole to remove nonspecifically bound protein. The His-tagged protein was eluted with 5 CV of elution buffer containing 50 mM sodium phosphate buffer (pH 7.5), 500 mM NaCl, 10 mM βME, 10% glycerol (v/v), 0.02% LMNG, and 300 mM imidazole. The eluate was loaded onto a HiLoad 16/600 Superdex 200 pg column (Cytiva) preequilibrated with SEC buffer containing 20 mM Bis Tris pH 6.5, 100 mM NaCl, 10 mM βME, 1 mM DTT, and 0.02% DDM, and purified by size-exclusion chromatography. The appropriate peak was pooled. Protein concentration was determined by measuring absorbance at 280 nm using a NanoDrop spectrophotometer (Thermo Fisher Scientific). Concentrations were calculated based on the protein’s theoretical extinction coefficient and molecular weight. Protein was aliquoted, flash frozen in liquid nitrogen and stored at −80 °C. Protein purity (>95%) was confirmed by SDS-PAGE with Coomassie Blue staining.

### NBD SM Activity Assay

C12-NBD sphingomyelin (NBD SM, 810219P; Avanti Polar Lipids) was used to determine activity by adapting previously published method.^44^ NBD SM and phosphatidylserine (PS, 840036P; Avanti Polar Lipids) were dried under nitrogen gas. Dried lipids were resuspended in assay buffer A containing 200 mM Tris pH 7.5, 300 mM NaCl, 10 mM MgCl_2_ and 0.2% Triton X-100 (Research Products International) and sonicated to make mixed micelles. Final assay conditions were: 100 mM Tris pH 7.5, 150 mM NaCl, 5 mM MgCl_2_ and 0.1% Triton X-100.

Purified human nSMase2 ΔIns (60 pM) was incubated with 2 mM mixed micelles that had a final concentration of 3 mol % NBD SM and 10 mol % PS. The reaction was incubated at 30 °C for 1 h, then stopped by the addition of a 2:1 chloroform methanol solution and extracted by a modified Bligh–Dyer method.^48^ The organic layer was pipetted into a clean glass tube, dried under nitrogen gas, and resuspended in HPLC buffer containing 1 mM ammonium formate (Acros Organics) and 0.2% formic acid (Fisher Chemical) in HPLC grade methanol.^55^ A volume of 10 μL of the resuspended mixture was loaded onto the HPLC. To quantify the hydrolyzed C12-NBD ceramide, a standard curve was used to convert the area under the curve to picomoles of ceramide generated.

### Mammalian Cell Transfection

HEK293T cells were passaged and seeded at 0.6 × 10^6^ cells per well in a 6-well plate, then cultured in DMEM supplemented with 10% fetal bovine serum (FBS) at 37 °C and 5% CO_2_ for 24 h. When the cells reached approximately 60–70% confluency, they were transfected with 2.5 μg DNA using the Lipofectamine 3000 reagent (Thermo Fisher Scientific) according to the manufacturer’s protocol, with OPTI-MEM as the transfection medium. Cells were grown at 37 °C and 5% CO_2_ for 48 h post-transfection.

### Mammalian Cell Harvesting

To harvest cells, the media were aspirated, and transfected HEK cells were washed twice with 4 mL of PBS. Cells were then scraped in PBS and transferred to microcentrifuge tubes. The cells were pelleted by centrifugation at 98 × g for 5 min, and the supernatant was aspirated. The cell pellet was resuspended in lysis buffer containing 50 mM Tris, pH 7.4, 5 mM EDTA, 0.2% Triton X-100, and 10 mM protease inhibitors pepstatin A (Goldbio), aprotinin (Goldbio) and leupeptin hemisulfate (Goldbio) and incubated on ice for 15 min. Following sonication, the lysate was centrifuged at 573 × g for 10 min. The resulting supernatant was transferred to a fresh microcentrifuge tube and kept on ice. Protein concentration was determined using a Bradford assay (Thermofisher Scientific), with bovine serum albumin as a standard.

### Western Blotting

Equal amounts of total protein (10 μg per sample) were resolved on 10% SDS-PAGE gels and transferred to nitrocellulose membranes (Bio-Rad) in Tris–glycine buffer for 1 h at 10 V. Membranes were blocked with 5% nonfat dry milk in TBST for 1 h at room temperature, then incubated overnight at 4 °C with primary antibodies against V5 (Thermo Fisher Scientific Cat# R960-25, RRID:AB_2556564) and β-actin (Sigma-Aldrich Cat# A5441, RRID:AB_476744)). After washing, membranes were probed with HRP-conjugated antimouse IgG antibodies (Jackson ImmunoResearch Laboratories Cat# 111-035-003, RRID:AB_2313567) for 1 h at room temperature. Protein bands were visualized using an enhanced chemiluminescence (ECL) substrate and imaged on a GE Amersham Imager 680.

### FRET Activity Assay

Phosphatidylserine (PS; 840036P, Avanti Polar Lipids) and phosphatidic acid (PA; 840875, Avanti Polar Lipids) were dried under nitrogen gas and resuspended in assay buffer B1 composed of 0.106% triton X-100, 117.64 mM Tris pH 7.5, 176.47 mM NaCl for + PS conditions, and buffer B2 composed of 0.118% triton X-100, 117.64 mM Tris pH 7.5, 176.47 mM for – PS conditions. Unless otherwise noted, the assays were performed with PCK ± 10 mol % PS. The PCK FRET substrate was added to the lipid solution, which was then sonicated for 2 min with intermittent vortexing to facilitate micelle formation. In a 96-well plate (3904; Corning), recombinant protein, cell lysate, or buffer control were added to each well, followed by the optional addition of 5 mM Mg^2+^ and finally the micellar FRET probe solution. For the dose–response studies for allosteric PS or PA activation, micelles contained 0–18 mol % PS (0, 0.81, 1.62, 3.25, 6.5, 13, 18) or 0–3.2 mol % PA (0, 0.2, 0.4, 0.8, 1.6, 3.2). The total micelle concentration (1.7 mM) was held constant by compensatory adjustment of Triton X-100 concentration.

The final working conditions in each well were 0.1% Triton X-100, 150 mM NaCl, 100 mM Tris pH 7.4, 10 mM DTT, and 1 μM PCK probe unless otherwise indicated. Fluorescence was measured over time using a SpectraMax M2 plate reader (Molecular Devices) using a bottom-read mode with an excitation wavelength of 485 nm for fluorescein (FAM) and emission wavelengths of 535 and 625 nm for FAM and BODIPY, respectively.

### GW4869 Inhibitor Testing

Recombinant nSMase2 ΔIns (22.5 nM) or buffer control was incubated with varying concentrations of GW4869 or DMSO control + vehicle (5 μL of 5% methanesulfonic acid was added to 50 μL stock of GW4869 and DMSO control) for 15 min at room temperature. The PCK substrate and 5 mol % PS were resuspended in assay buffer C1 (composed of 0.18% triton X-100, 200 mM Tris pH 7.5, 300 mM NaCl, 10 mM Mg^2+^), whereas the PCK substrate only was resuspended in assay buffer C2 (composed of 0.2% triton X-100, 200 mM Tris pH 7.5, 300 mM NaCl, 10 mM Mg^2+^). The micelle containing solution was then added to a final concentration of 0.1% Triton X-100, 150 mM NaCl, 100 mM Tris pH 7.5, 5 mM Mg^2+^, 4 μM PCK probe, 10 mM DTT ± 5 mol % PS. Fluorescent readings were obtained as described in the activity assay section. In addition to FAM emission, BODIPY TR emission was measured at 625 nm, and the donor-to-acceptor fluorescence ratio was calculated. Background signals from buffer-only control samples lacking enzyme were processed identically and subtracted from experimental values. Slopes (fluorescence change over time) were then calculated, and relative activity was determined by normalizing to the DMSO plus vehicle control, set as 100% activity.

## Results

Since there is a need to develop new assays to measure nSMase2 activity for inhibitor testing, we developed a FRET assay using the PCK probe (Figure 1). Prior work showed that the PCK probe was hydrolyzed by aSMase, but not nSMase2.^50^

To test whether nSMase2 could hydrolyze PCK, we purified recombinant human nSMase2 ΔIns (Figure 2A) from *E. coli* using sequential nickel affinity purification and size exclusion chromatography (Figure 2B and Figure S1). Milligram quantities of protein with >95% purity were obtained (Figure 2B).) Recombinant nSMase2 exhibited catalytic activity with NBD SM as a substrate and showed characteristic activation by phosphatidylserine (PS) (Figure 2C). Notably the nSMase2 ΔIns protein purified here displayed activity on a per second range (2.5 s^–1^), which was 5 orders of magnitude higher than the previously characterized JX-CAT nSMase2 protein (0.07 h^–1^).^17^

**Figure 2:**
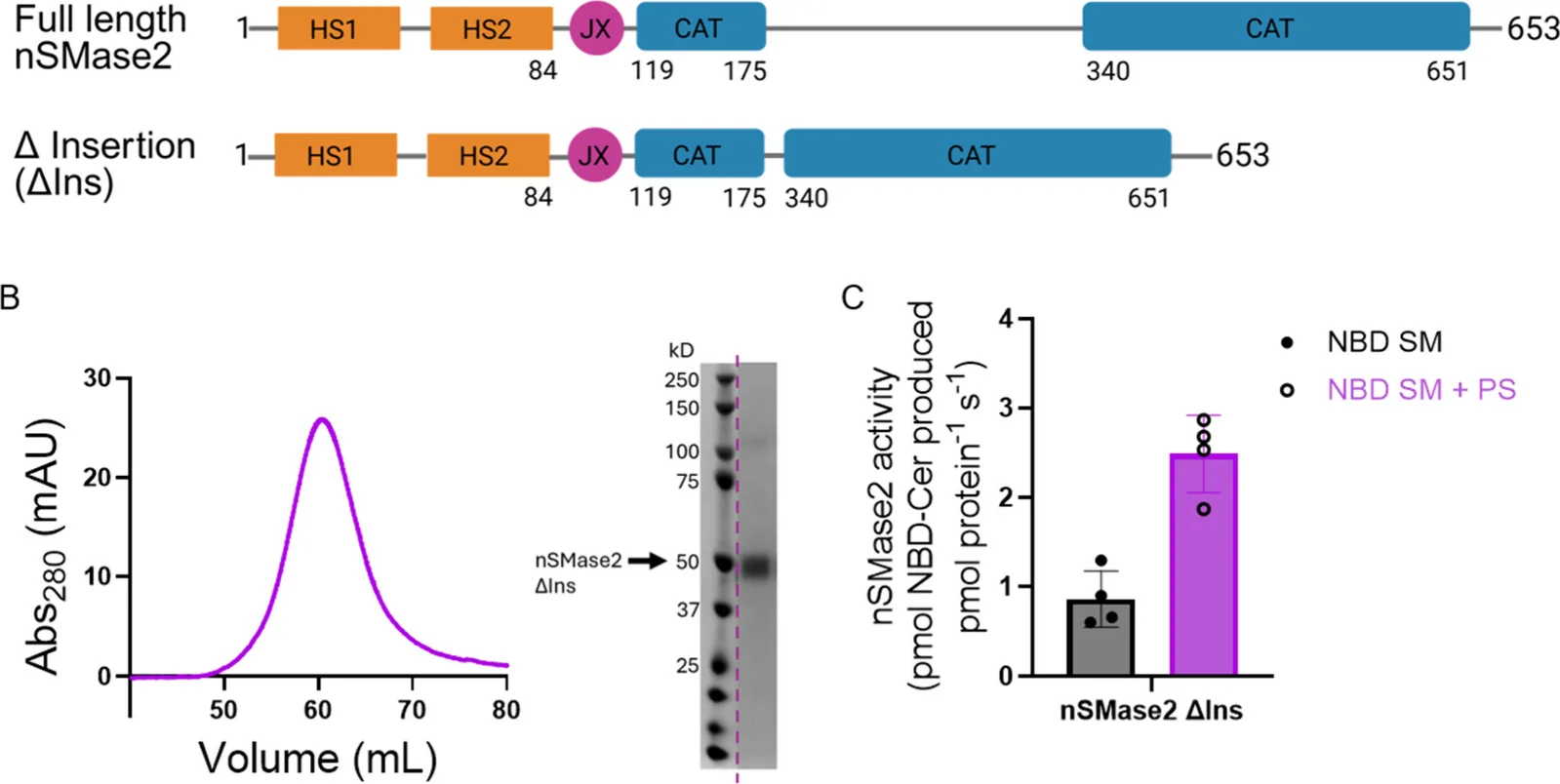
Purified human nSMase2 ΔIns is catalytically active against the native substrate sphingomyelin and allosterically activated by phosphatidylserine. (A) Domain architecture of human nSMase2 full length and ΔIns with deletion of residues 175-339. The domains consist of two hydrophobic segments (HS), juxtamembrane region (JX), and catalytic domain (CAT) with a large insertion region. (B) Human nSMase2 ΔIns, expressed in *E. coli*, was purified by Ni-NTA affinity and size exclusion chromatography (left). SDS PAGE with Coomassie blue staining confirms >95% purity (right). (C) Activity of recombinant nSMase2 ΔIns was tested using NBD SM ± 10 mol % phosphatidylserine (PS). The canonical lipid activator PS increased nSMase2 activity ∼ 3-fold. Data represent mean ± SD (*n* = 4).

To assess PCK as an nSMase2 substrate, recombinant nSMase2 was first used. Using a Triton X-100 mixed-micelle enzymatic assay with 1 μM PCK, the results showed linear increase in fluorescence at 535 nm over time with incubation with nSMase2 ΔIns and Mg^2+^ (Figure 3). For simplicity, only the fluorescence at 535 nm was used as the readout. Protein concentration linearity was determined (Figure S2C). As negative controls, reactions containing buffer with Mg^2+^ alone, nSMase2 without Mg^2+^ and an inactive point mutant N130A were included. None of the negative controls increased fluorescence. Together, these results indicate that PCK acts as a substrate of nSMase2.

**Figure 3:**
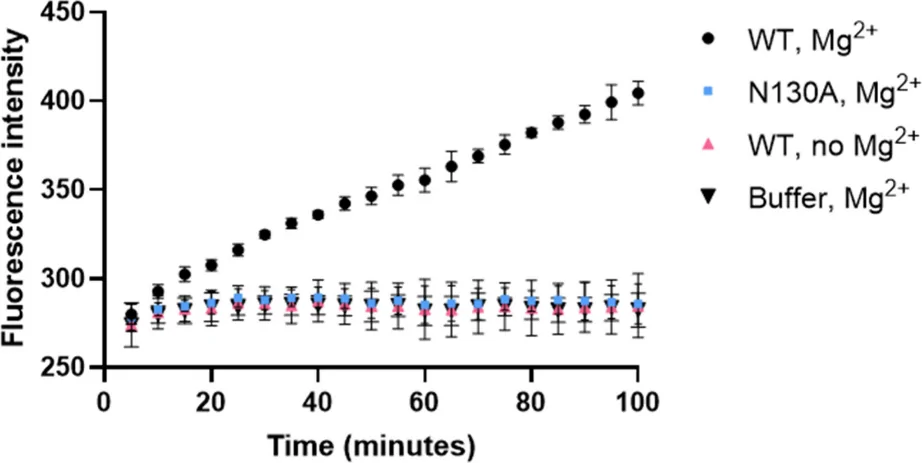
Recombinant human nSMase2 ΔIns hydrolyzes FRET sphingomyelin analogue PCK. Recombinant nSMase2 ΔIns (45 nM) was incubated with 1 μM PCK and 5 mM Mg^2+^ cofactor. Negative controls included buffer only, WT nSMase2 without Mg^2+^ and catalytically dead N130A nSMase2. Data represent mean ± SD (*n* = 3).

To determine whether nSMase2 hydrolysis of PCK was allosterically activated by anionic lipids, recombinant nSMase2 activity was measured under varying concentrations of PS (0–18 mol %) and PA (0–3.2 mol %) in a mixed micelle system with PCK. At 10 mol % PS, a 5-fold increase in activity was observed (Figure 4A). The results also showed that both PS and PA increased nSMase2 activity in a dose-dependent manner (Figure 4B, 4C). Hill coefficients of 2.4 for PS and 1.4 for PA were calculated and indicate positive cooperativity; these results are consistent with previous mutagenesis and kinetic data that suggested the presence of 2 PS and 1 PA binding sites in mouse nSMase2.^38,56^ Together, the results show that the FRET assay system with PCK probe demonstrates PS and PA allosteric activation, which are hallmarks of nSMase2 activity.

**Figure 4:**
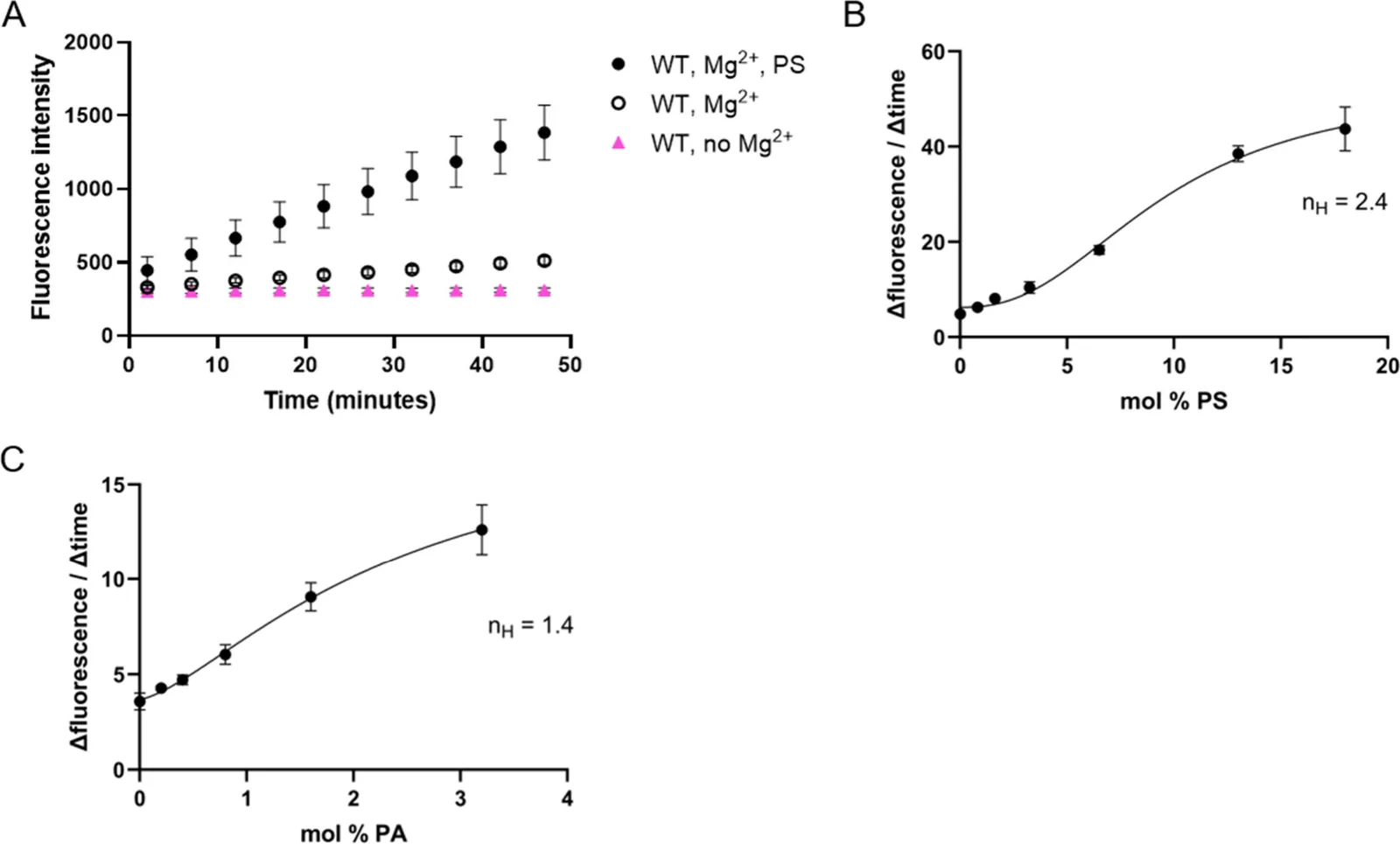
PCK shows dose-dependent allosteric activation of nSMase2 ΔIns by phosphatidylserine (PS) and phosphatidic acid (PA). (A) WT nSMase2 ΔIns (0.182 μM) was incubated with mixed micelles containing 10 mol % PS and 1 μM PCK. At 10 mol % PS, nSMase2 activity increased ∼ 5-fold compared to WT incubated with Mg^2+^ but without PS. Data represent mean ± SD (*n* = 3). (B) Micelles containing 0–18 mol % PS or (C) 0–3.2 mol % PA and 1 μM PCK were incubated with recombinant nSMase2 ΔIns. The total micelle concentration was held constant by compensatory adjustment of Triton X-100 concentration. Real-time fluorescence measurements were recorded, and initial rates (slopes of fluorescence vs time) were calculated and plotted. The resulting curves were fitted using the 4-parameter logistic equation in GraphPad Prism, yielding Hill coefficients (*n*_H_) of 2.4 for PS and 1.4 for PA. Data represent mean ± SD (*n* = 3).

To establish the lower detection limit, dynamic range, and fidelity of the FRET assay, we purified and evaluated the activity of mutants that have been previously demonstrated to have low activity. These mutants included the previously characterized mutations of residues R33, KRQR 45-48, R92 and R93 that are important for PS binding,^38^ the conserved residue N130 involved in Mg^2+^ coordination, and the residue D430 that is part of the universally conserved DK switch important for substrate recognition.^17^ Band intensities of Coomassie-stained wild-type and mutant nSMase2 loaded at the same total protein amount confirm comparable purity (Figure 5A). The activity results demonstrated that the probe clearly detected 25% activity in R92/93A, 10% activity in R33A Δ45-48, and 2% activity in D430A, with no activity detected in the catalytically dead mutant N130A relative to WT (Figure 5B). Together, we concluded that the FRET assay system with PCK probe is sensitive and can resolve weak activities across a panel of low activity mutants.

**Figure 5:**
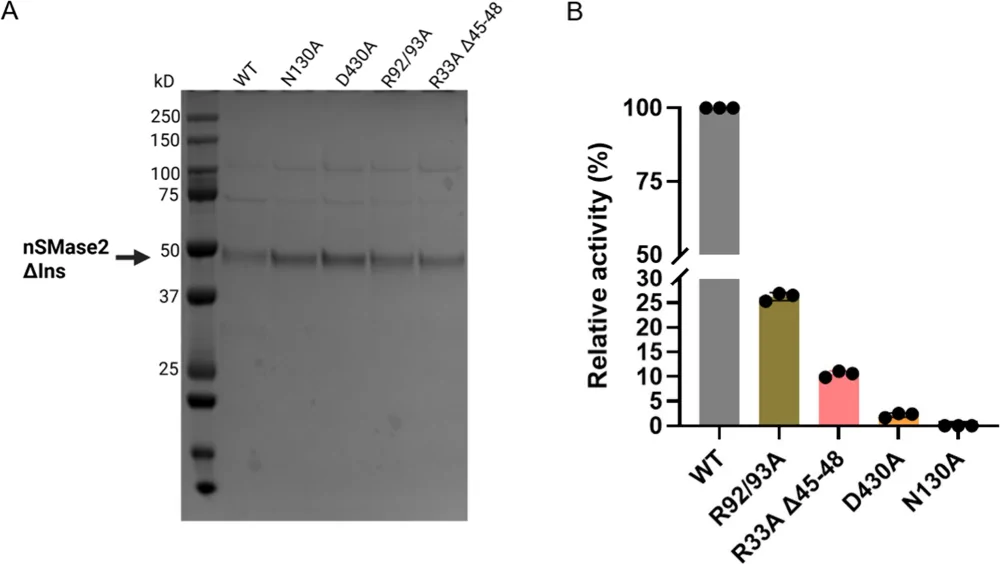
PCK detects low-level activity of mutant proteins. (A) SDS-PAGE with Coomassie blue staining of WT and mutant proteins confirms >95% purity and equal amount of protein (0.09 μM). (B) Relative activity of WT nSMase2 compared to mutants with low activity and catalytically dead N130A in the presence of 10 mol % PS. Reaction rates (slopes of fluorescence change over time) were calculated, and relative activity was determined by normalizing to WT (set as 100% activity). Data represent mean ± SD (*n* = 3).

Having demonstrated that the FRET PCK system captures allosteric lipid activation of nSMase2, the effect of the commonly used nSMase2 inhibitor GW4869 on nSMase2-mediated FRET probe hydrolysis was assessed. GW4869 is noncompetitive with the substrate SM but competitive with PS.^39^ nSMase2 ΔIns or buffer was incubated with 0–33 μM GW4869 or vehicle control (DMSO + methanesulfonic acid) for 15 min before the addition of mixed micelles containing 4 μM PCK ± 5 mol % PS. A dose dependent inhibition of nSMase2 activity by GW4869 in the absence and presence of PS was observed, with calculated IC_50_ values of 1.2 μM and 1.5 μM (Figure 6). The calculated values are similar to prior published IC_50_ values around 1 μM.^17,39^ This indicates that the FRET probe effectively captures nSMase2 inhibition by GW4869.

**Figure 6:**
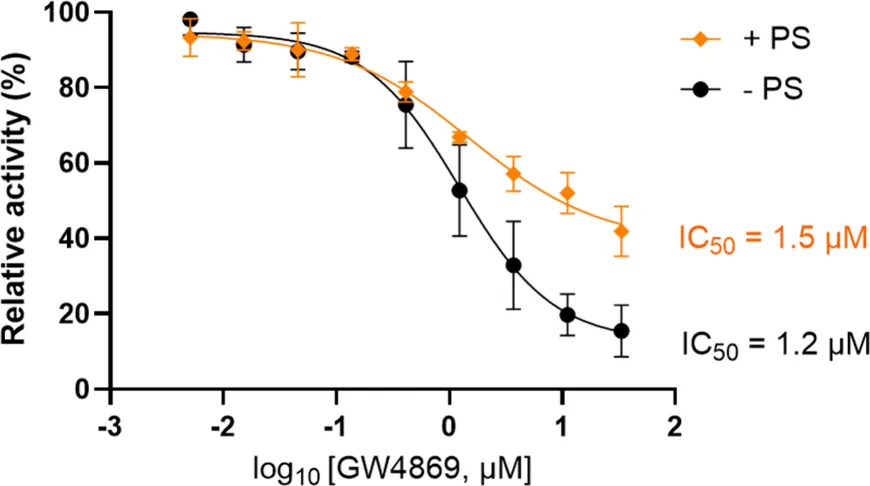
PCK captures dose-dependent inhibition of nSMase2 by inhibitor GW4869 in the presence and absence of PS. Recombinant WT nSMase2 ΔIns (22.5 nM) or buffer-only control was incubated with increasing concentrations of inhibitor GW4869 (0–33 μM). A micellar solution containing 4 μM PCK substrate ± 5 mol % PS was added, and real-time fluorescence measurements were obtained for each condition, including DMSO plus vehicle control without inhibitor. Reaction rates were determined from the slopes of donor: acceptor emission change over time. Background donor-to-acceptor ratios from buffer-only controls lacking enzyme were subtracted from experimental traces prior to slope fitting and normalization. Relative activity was determined by normalizing to the DMSO plus vehicle control (set as 100% activity). The resulting curves were fitted using the 4-parameter logistic model in GraphPad Prism, yielding IC_50_ values of 1.2 μM (− PS) and 1.5 μM (+ PS). Data represent mean ± SD (*n* = 3).

While our original goal was to establish an assay for nSMase2 compatible with HTS, which we demonstrated using recombinant purified protein, we next sought to establish if the assay was compatible with cell lysates. Toward this, V5-tagged full length nSMase2 was overexpressed in HEK293T cells by transient transfection. As negative controls, the inactive point mutant N130A and empty vector were included. To ensure that the transfection was successful, Western blot studies were performed to probe for the V5 tag, and β actin was used as a loading control (Figure 7A). For the activity assay, 5 μg of cell lysates were incubated with a micellar solution containing 1 μM PCK with 10 mol % PS. An increase in fluorescence over time was observed in WT nSMase2, but not in the negative controls (Figure 7B).

**Figure 7:**
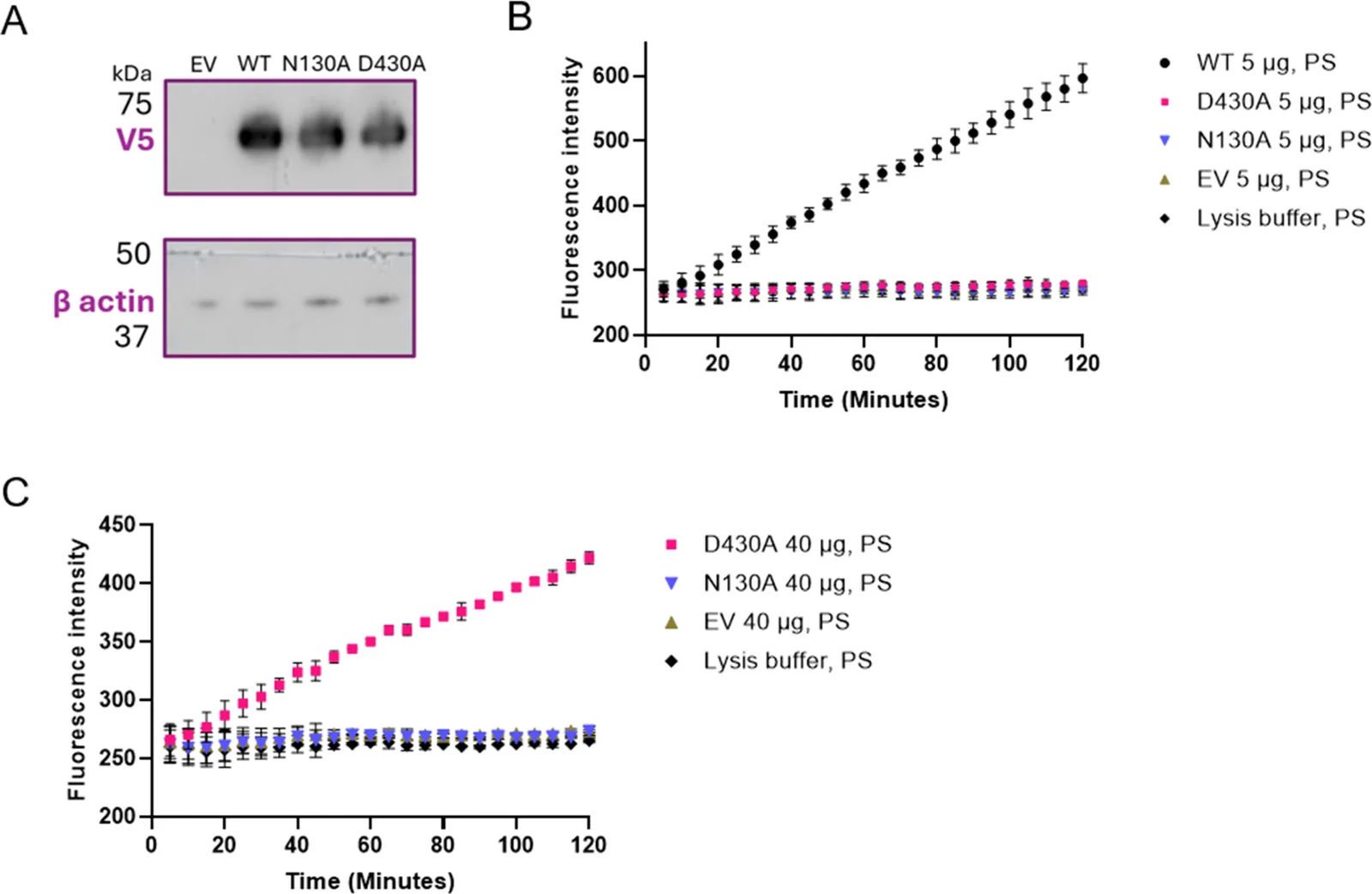
PCK is hydrolyzed by wild-type and mutant full length nSMase2 overexpressed in HEK cells. (A) Representative Western blot showing expression of V5-tagged wild-type (WT) and mutant nSMase2 proteins, probed with anti-V5 and β-actin as a loading control. (B) Cell lysates containing 5 μg of WT nSMase2 hydrolyze the FRET substrate. Data represent mean ± SD (*n* = 3). (C) Lysates with 40 μg of the low-activity mutant D30A show substrate hydrolysis, while the catalytically dead mutant N130A does not hydrolyze the substrate. Data represent mean ± SD (*n* = 2).

Next, the activity of a low activity mutant, D430A, which was shown to have 1% activity compared to wild-type using recombinant protein^17^ was tested to better assess the sensitivity of the PCK assay system. At 5 μg of D430A cell lysates, barely any activity was detected (Figure 7B). However, at 40 μg of cell lysates, activity was observed (Figure 7C). We concluded that PCK was sensitive enough to detect nSMase2 activity from cell lysates in both wild type and in a low activity mutant when assessed from cell lysates (Figure 7B, 7C).

In conclusion, to explore nSMase2 as a potential drug target and address the need for efficient tools to evaluate its activity, fluorescent substrate analogues were developed. A plate-based assay system using PCK FRET probe was established for high-throughput inhibitor screening. This system was shown to be both sensitive and reliable, detecting activity from wild-type nSMase2, as well as low-activity mutants. Furthermore, the assay’s utility in inhibitor screening was validated using commonly used nSMase2 inhibitor, GW4869, which demonstrated inhibitory effects.

## Discussion

This study successfully demonstrates the adaptation of the FRET-based SM analogue PCK, originally developed for aSMase, as a sensitive real-time tool for monitoring the catalytic activity of nSMase2. The results demonstrate that PCK can detect nSMase2 activity *in vitro,* using either recombinant protein or lysates from nSMase2-overexpressing cells. This addresses a critical gap in enzymology and drug discovery assays for nSMase2.

An increase in fluorescence over time was observed when PCK was incubated with WT nSMase2 but not with catalytically dead N130A mutant (Figure 3 and 7B), demonstrating that PCK is a substrate for nSMase2 and that loss of FRET signal upon enzymatic hydrolysis provides a sensitive readout of enzymatic function. We note that we did not conduct a competition assay with native SM and the FRET substrate due to the high *K*_*M*_ of PCK in Triton X-100 mixed micelles and the fact that the first part of a *K*_*M*_ curve is near linear at the low concentration of PCK under our assay conditions. However, PCK was able to capture allosteric activation by phosphatidylserine (PS) and phosphatidic acid (PA) (Figure 4), detect comparable low activity (2% of WT) for the D430A mutant compared to radiolabeled SM (1% of WT), as well as inhibition by the commonly used nSMase2 inhibitor GW4869 (Figure 6). Thus, these findings are consistent with previous studies using radiolabeled substrates^17,38,39^ while offering a nonradioactive alternative.

This approach widens the tool kit for nSMase2 assays by providing a real-time method that can be used in medium- or high-throughput assays and enzymology studies. Additionally, since it is a plate-based method and abrogates the need for organic extraction, it streamlines the assay process and makes it simpler and more efficient. PCK was suitable for kinetic studies and inhibitor testing.

Although GW4869 is the most commonly used nSMase2 inhibitor, its poor solubility restricts its potential in drug development, underscoring the need for more potent inhibitors with improved physicochemical properties. The successful purification of recombinant human nSMase2 and its robust hydrolysis of the PCK FRET probe (Figure 3) demonstrate a reliable platform for evaluating inhibitor efficacy, supporting its use in high-throughput screening to identify novel, more soluble compounds.

We identified a limitation to the use of PCK when we attempted to assess endogenous nSMase2 activity from MCF-7 cell lysates, which is very low in most cells in culture (data not shown). The results suggest that the PCK probe may also be hydrolyzed by other phospholipases and N-SMases that are active at pH 7.5 (data not shown). This cross-reactivity limits the probe’s utility in complex cellular environments. Thus, PCK is not suitable for robust quantification of endogenous nSMase2 activity in untransfected cell lysates. Future development of more selective probes that better discriminate nSMase2 activity may address this issue. aSMase can contribute to PCK hydrolysis in lysates. Future work will be needed to systematically dissect the contributions of different SMase isoforms under lysate conditions in different pH conditions. Such work is beyond the scope of the present study, which focuses on establishing a real-time high throughput compatible assay for nSMase2. Additionally, PCK’s bulky fluorophores are a limitation. However, our study focuses on defining the conditions under which nSMase2 can utilize PCK rather than redesigning the probe.

One technical consideration for inhibitor screening is that the fluorescent signal occurs at wavelengths where some small molecules may also fluoresce or absorb. However, this can be addressed by measuring the ratio of donor-to-acceptor emission to control for compound interference, as we did in Figure 6. Additionally, by using the fluorescence slope, which is largely independent of spectral interference, instead of a single end point readout, one could reduce issues caused by spectrally interfering inhibitors. Furthermore, implementing a secondary high-throughput counter-screen would further validate hit specificity.

Finally, and importantly, this work establishes that nSMase2 activity can be reliably measured by using a plate reader-based fluorescence assay. While other assays use the native, unmodified substrate SM coupled to organic extraction and radioactivity quantitation, the current method is more accessible and practical for laboratories without specialized experimental set up, less laborious, and offers real-time measurements. The method offers a valuable tool that expands how nSMase2 and similar enzymes can be studied experimentally.

## Supplementary Material



## Notes

Figure S1. Full image of the SDS PAGE gel stained with Coomassie blue used for Figure 1B (right). Figure S2. Assay parameters time and enzyme linearity. Figure S3. Full, uncropped image of Western blot used for Figure 6A (PDF)

## Notes

nSMase2: UniProt ID Q9NY59; aSMase: UniProt ID P17405.

## References

[1] Hannun Y. A. (1996). Functions
of ceramide in coordinating cellular responses to stress. Science.

[2] Kolesnick R. N., Goni F. M., Alonso A. (2000). Compartmentalization
of ceramide
signaling: physical foundations and biological effects. J. Cell Physiol.

[3] Schneider P. B., Kennedy E. P. (1967). Sphingomyelinase
in normal human spleens and in spleens
from subjects with Niemann-Pick disease. J.
Lipid Res..

[4] Rao B. G., Spence M. W. (1976). Sphingomyelinase Activity at Ph 7.4
in Human-Brain
and a Comparison to Activity at Ph 5.0. J. Lipid
Res..

[5] Nilsson A. (1969). The presence
of spingomyelin- and ceramide-cleaving enzymes in the small intestinal
tract. Biochim. Biophys. Acta.

[6] Duan R. D., Nilsson A. (1997). Purification
of a newly identified alkaline sphingomyelinase
in human bile and effects of bile salts and phosphatidylcholine on
enzyme activity. Hepatology.

[7] Marchesini N., Hannun Y. A. (2004). Acid and neutral
sphingomyelinases: roles and mechanisms
of regulation. Biochem Cell Biol..

[8] Kim M. Y., Linardic C., Obeid L., Hannun Y. (1991). Identification of sphingomyelin
turnover as an effector mechanism for the action of tumor necrosis
factor alpha and gamma-interferon. Specific role in cell differentiation. J. Biol. Chem..

[9] Rutkute K., Asmis R. H., Nikolova-Karakashian M. N. (2007). Regulation
of neutral
sphingomyelinase-2 by GSH: a new insight to the role of oxidative
stress in aging-associated inflammation. J.
Lipid Res..

[10] Karakashian A. A., Giltiay N. V., Smith G. M., Nikolova-Karakashian M. N. (2004). Expression
of neutral sphingomyelinase-2 (NSMase-2) in primary rat hepatocytes
modulates IL-beta-induced JNK activation. FASEB
J..

[11] Levy M., Castillo S. S., Goldkorn T. (2006). nSMase2 activation and trafficking
are modulated by oxidative stress to induce apoptosis. Biochem. Biophys. Res. Commun..

[12] Cogolludo A., Moreno L., Frazziano G., Moral-Sanz J., Menendez C., Castaneda J., Gonzalez C., Villamor E., Perez-Vizcaino F. (2008). Activation
of neutral sphingomyelinase is involved
in acute hypoxic pulmonary vasoconstriction. Cardiovasc. Res..

[13] Hofmann K., Tomiuk S., Wolff G., Stoffel W. (2000). Cloning and characterization
of the mammalian brain-specific, Mg2+-dependent neutral sphingomyelinase. Proc. Natl. Acad. Sci. U. S. A..

[14] Uhlen M., Fagerberg L., Hallstrom B. M., Lindskog C., Oksvold P., Mardinoglu A., Sivertsson A., Kampf C., Sjostedt E., Asplund A. (2015). Proteomics. Tissue-based map of the human proteome. Science.

[15] Marchesini N., Osta W., Bielawski J., Luberto C., Obeid L. M., Hannun Y. A. (2004). Role for mammalian
neutral sphingomyelinase 2 in confluence-induced
growth arrest of MCF7 cells. J. Biol. Chem..

[16] Hostetler K. Y., Yazaki P. J. (1979). The subcellular localization of neutral sphingomyelinase
in rat liver. J. Lipid Res..

[17] Airola M. V., Shanbhogue P., Shamseddine A. A., Guja K. E., Senkal C. E., Maini R., Bartke N., Wu B. X., Obeid L. M., Garcia-Diaz M. (2017). Structure of human nSMase2 reveals an interdomain
allosteric activation mechanism for ceramide generation. Proc. Natl. Acad. Sci. U. S. A..

[18] Stoffel W., Jenke B., Block B., Zumbansen M., Koebke J. (2005). Neutral sphingomyelinase 2 (smpd3)
in the control of
postnatal growth and development. Proc. Natl.
Acad. Sci. U. S. A..

[19] Piacentino M. L., Hutchins E. J., Andrews C. J., Bronner M. E. (2022). Temporal changes
in plasma membrane lipid content induce endocytosis to regulate developmental
epithelial-to-mesenchymal transition. Proc.
Natl. Acad. Sci. U. S. A..

[20] Trajkovic K., Hsu C., Chiantia S., Rajendran L., Wenzel D., Wieland F., Schwille P., Brugger B., Simons M. (2008). Ceramide triggers budding
of exosome vesicles into multivesicular endosomes. Science.

[21] Choezom, D. ; Gross, J. C. Neutral sphingomyelinase 2 controls exosome secretion by counteracting V-ATPase-mediated endosome acidification. J. Cell Sci. 2022, 135 (5). 10.1242/jcs.259324. PMC8919340 35050379

[22] Asai H., Ikezu S., Tsunoda S., Medalla M., Luebke J., Haydar T., Wolozin B., Butovsky O., Kugler S., Ikezu T. (2015). Depletion of microglia and inhibition of exosome synthesis halt tau
propagation. Nat. Neurosci.

[23] Dinkins M. B., Dasgupta S., Wang G., Zhu G., Bieberich E. (2014). Exosome reduction
in vivo is associated with lower amyloid plaque load in the 5XFAD
mouse model of Alzheimer’s disease. Neurobiol
Aging.

[24] Kim W. J., Okimoto R. A., Purton L. E., Goodwin M., Haserlat S. M., Dayyani F., Sweetser D. A., McClatchey A. I., Bernard O. A., Look A. T. (2008). Mutations
in the neutral
sphingomyelinase gene SMPD3 implicate the ceramide pathway in human
leukemias. Blood.

[25] Lallemand T., Rouahi M., Swiader A., Grazide M. H., Geoffre N., Alayrac P., Recazens E., Coste A., Salvayre R., Negre-Salvayre A. (2018). nSMase2 (Type 2-Neutral Sphingomyelinase) Deficiency
or Inhibition by GW4869 Reduces Inflammation and Atherosclerosis in
Apoe­(−/−) Mice. Arterioscler Thromb
Vasc Biol..

[26] Filippov V., Song M. A., Zhang K., Vinters H. V., Tung S., Kirsch W. M., Yang J., Duerksen-Hughes P. J. (2012). Increased
ceramide in brains with Alzheimer’s and other neurodegenerative
diseases. J. Alzheimers Dis.

[27] Gu L., Huang B., Shen W., Gao L., Ding Z., Wu H., Guo J. (2013). Early activation of
nSMase2/ceramide pathway in astrocytes
is involved in ischemia-associated neuronal damage via inflammation
in rat hippocampi. J. Neuroinflammation.

[28] Takahashi R. H., Milner T. A., Li F., Nam E. E., Edgar M. A., Yamaguchi H., Beal M. F., Xu H., Greengard P., Gouras G. K. (2002). Intraneuronal Alzheimer abeta42 accumulates in multivesicular
bodies and is associated with synaptic pathology. Am. J. Pathol..

[29] Dinkins M. B., Enasko J., Hernandez C., Wang G., Kong J., Helwa I., Liu Y., Terry A. V., Bieberich E. (2016). Neutral Sphingomyelinase-2
Deficiency Ameliorates Alzheimer’s
Disease Pathology and Improves Cognition in the 5XFAD Mouse. J. Neurosci..

[30] Tohumeken S., Deme P., Yoo S. W., Gupta S., Rais R., Slusher B. S., Haughey N. J. (2023). Neuronal
deletion of nSMase2 reduces
the production of Abeta and directly protects neurons. Neurobiol Dis.

[31] Grimm M. O., Grimm H. S., Patzold A. J., Zinser E. G., Halonen R., Duering M., Tschape J. A., De Strooper B., Muller U., Shen J. (2005). Regulation of cholesterol
and sphingomyelin metabolism by amyloid-beta and presenilin. Nat. Cell Biol..

[32] Jana A., Pahan K. (2010). Fibrillar amyloid-beta-activated
human astroglia kill primary human
neurons via neutral sphingomyelinase: implications for Alzheimer’s
disease. J. Neurosci..

[33] Han X., Rozen S., Boyle S. H., Hellegers C., Cheng H., Burke J. R., Welsh-Bohmer K. A., Doraiswamy P. M., Kaddurah-Daouk R. (2011). Metabolomics in early Alzheimer’s
disease: identification of altered plasma sphingolipidome using shotgun
lipidomics. PLoS One.

[34] Cutler R. G., Kelly J., Storie K., Pedersen W. A., Tammara A., Hatanpaa K., Troncoso J. C., Mattson M. P. (2004). Involvement of oxidative
stress-induced abnormalities in ceramide and cholesterol metabolism
in brain aging and Alzheimer’s disease. Proc. Natl. Acad. Sci. U. S. A..

[35] Huang M., Stremlau M., Zavras J., Zivko C., Thomas A. G., Pietri P., Machairaki V., Slusher B. S. (2025). Neutral sphingomyelinase
2: A promising drug target for CNS disease. Adv. Pharmacol..

[36] Tani M., Hannun Y. A. (2007). Analysis of membrane topology of
neutral sphingomyelinase
2. FEBS Lett..

[37] Marchesini N., Luberto C., Hannun Y. A. (2003). Biochemical
properties of mammalian
neutral sphingomyelinase 2 and its role in sphingolipid metabolism. J. Biol. Chem..

[38] Wu B. X., Clarke C. J., Matmati N., Montefusco D., Bartke N., Hannun Y. A. (2011). Identification of
novel anionic phospholipid
binding domains in neutral sphingomyelinase 2 with selective binding
preference. J. Biol. Chem..

[39] Luberto C., Hassler D. F., Signorelli P., Okamoto Y., Sawai H., Boros E., Hazen-Martin D. J., Obeid L. M., Hannun Y. A., Smith G. K. (2002). Inhibition of tumor
necrosis factor-induced cell death
in MCF7 by a novel inhibitor of neutral sphingomyelinase. J. Biol. Chem..

[40] Pavlic A., Poelman H., Wasilewski G., Wichapong K., Lux P., Maassen C., Lutgens E., Schurgers L. J., Reutelingsperger C. P., Nicolaes G. A. F. (2023). Inhibition of
Neutral Sphingomyelinase
2 by Novel Small Molecule Inhibitors Results in Decreased Release
of Extracellular Vesicles by Vascular Smooth Muscle Cells and Attenuated
Calcification. Int. J. Mol. Sci..

[41] Figuera-Losada M., Stathis M., Dorskind J. M., Thomas A. G., Bandaru V. V., Yoo S. W., Westwood N. J., Rogers G. W., McArthur J. C., Haughey N. J. (2015). Cambinol,
a novel inhibitor of neutral sphingomyelinase
2 shows neuroprotective properties. PLoS One.

[42] Liu, B. ; Hannun, Y. A. [18] Sphingomyelinase assay using radiolabeled substrate. In Methods in enzymology; Elsevier, 2000; Vol. 311, pp 164–167. 10563321 10.1016/s0076-6879(00)11077-8

[43] Loidl A., Claus R., Deigner H. P., Hermetter A. (2002). High-precision
fluorescence assay for sphingomyelinase activity of isolated enzymes
and cell lysates. J. Lipid Res..

[44] Nikolova-Karakashian M. (2021). Methods to
Characterize Synthesis and Degradation of Sphingomyelin at the Plasma
Membrane and Its Impact on Lipid Raft Dynamics. Methods Mol. Biol..

[45] Nusshold C., Uellen A., Bernhart E., Hammer A., Damm S., Wintersperger A., Reicher H., Hermetter A., Malle E., Sattler W. (2013). Endocytosis and intracellular processing
of BODIPY-sphingomyelin by murine CATH.a neurons. Biochim. Biophys. Acta.

[46] Yi J., Qi B., Yin J., Li R., Chen X., Hu J., Li G., Zhang S., Zhang Y., Yang M. (2023). Molecular basis for
the catalytic mechanism of human neutral sphingomyelinases 1 (hSMPD2). Nat. Commun..

[47] Zhou M., Diwu Z., Panchuk-Voloshina N., Haugland R. P. (1997). A stable nonfluorescent
derivative of resorufin for the fluorometric determination of trace
hydrogen peroxide: applications in detecting the activity of phagocyte
NADPH oxidase and other oxidases. Anal. Biochem..

[48] Bligh E. G., Dyer W. J. (1959). A rapid method of total lipid extraction and purification. Can. J. Biochem Physiol.

[49] Pinkert T., Furkert D., Korte T., Herrmann A., Arenz C. (2017). Amplification
of a FRET Probe by Lipid-Water Partition for the Detection of Acid
Sphingomyelinase in Live Cells. Angew. Chem.,
Int. Ed. Engl..

[50] Kappe C., Mohamed Z. H., Naser E., Carpinteiro A., Arenz C. (2020). A Novel Visible Range FRET Probe
for Monitoring Acid Sphingomyelinase
Activity in Living Cells. Chemistry.

[51] Mohamed Z. H., Rhein C., Schmid B., Tripal P., Kornhuber J., Arenz C. (2021). Synthesis and characterization of a new two photon excitable acid
sphingomyelinase FRET probe. Bioorg. Med. Chem..

[52] Mohamed Z. H., Rhein C., Saied E. M., Kornhuber J., Arenz C. (2018). FRET probes for measuring sphingolipid
metabolizing enzyme activity. Chem. Phys. Lipids.

[53] Lakowicz, J. R. Principles of Fluorescence Spectroscopy; Springer, 2006. 10.1007/978-0-387-46312-4.

[54] Ruhling M., Kersting L., Wagner F., Schumacher F., Wigger D., Helmerich D. A., Pfeuffer T., Elflein R., Kappe C., Sauer M. (2024). Trifunctional sphingomyelin
derivatives enable nanoscale resolution of sphingomyelin turnover
in physiological and infection processes via expansion microscopy. Nat. Commun..

[55] Lee A. H., Snider J. M., Moorthi S., Coant N., Trayssac M., Canals D., Clarke C. J., Luberto C., Hannun Y. A. (2024). A comprehensive
measure of Golgi sphingolipid flux using NBD C(6)-ceramide: evaluation
of sphingolipid inhibitors. J. Lipid Res..

[56] Wu B. X., Clarke C. J., Hannun Y. A. (2010). Mammalian neutral sphingomyelinases:
regulation and roles in cell signaling responses. Neuromolecular Med..

