# Supplementary material for: Development of a FRET-Based Assay for Human Neutral Sphingomyelinase 2
Source: Biochemistry. 2026 Aug 7;65(16):2548–56. doi: 10.1021/acs.biochem.6c00322 (PMC13492263; doi:10.1021/acs.biochem.6c00322)
Supplement: Supplementary file 1 [file bi6c00322_si_001.pdf]

**Supporting information for**

# Development of a FRET-based assay for human neutral sphingomyelinase 2

Khalayi Martha Aywa[1,2], Christian Kappe[3], Botheina Ghandour[2], Shujuan Gao[1], Christoph Arenz[3*],

Yusuf A. Hannun[1,2*] and Michael V. Airola[1,2*]

[1] Department of Biochemistry and Cell Biology, Stony Brook University, Stony Brook NY 11794, USA

[2] Stony Brook University Cancer Center, Stony Brook, NY 11794, USA

[3] Institute of Chemistry Humboldt-Universität zu Berlin Brook-Taylor-Strasse 2, 12489 Berlin Germany

*Corresponding authors: arenzchr@hu-berlin.de, yusuf.hannun@stonybrookmedicine.edu, michael.airola@stonybrook.edu

Table of Contents

Figure S1. Full image of the SDS PAGE gel stained with Coomassie blue used for Figure 1B (right).

Figure S2. Assay parameters time and enzyme linearity.

Figure S3. Full, uncropped image of Western blot used for Figure 6A.

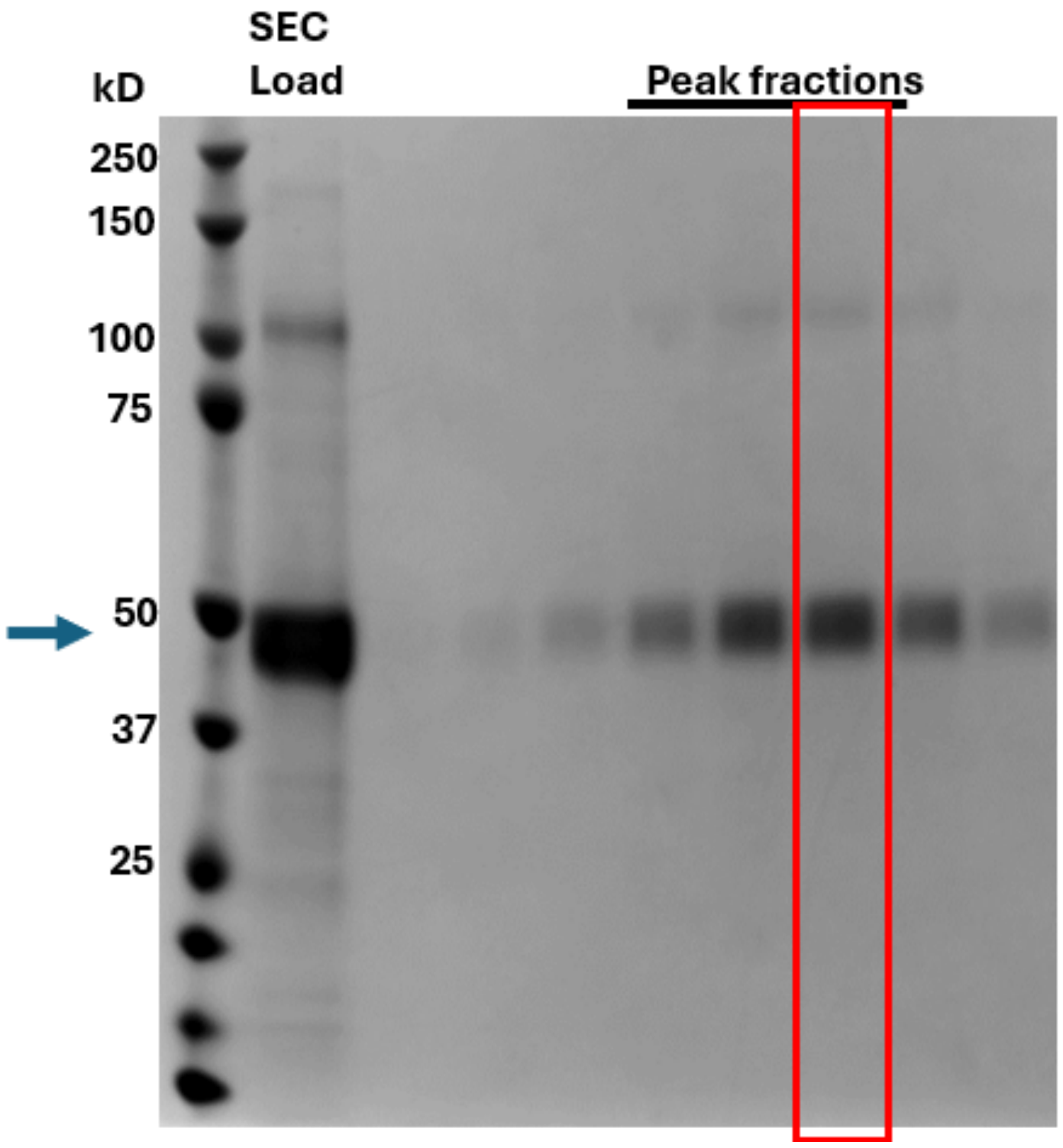


**Figure S1. Full, uncropped image of the SDS PAGE gel stained with Coomassie blue used for Figure 1B (right).** Red box outlines region presented in the main figure. Lane order: 1 = molecular weight marker, 2 = size exclusion column load, 3 – 10 = size exclusion fractions. Image was captured using Bio-Rad Gel Doc™ EZ imager.

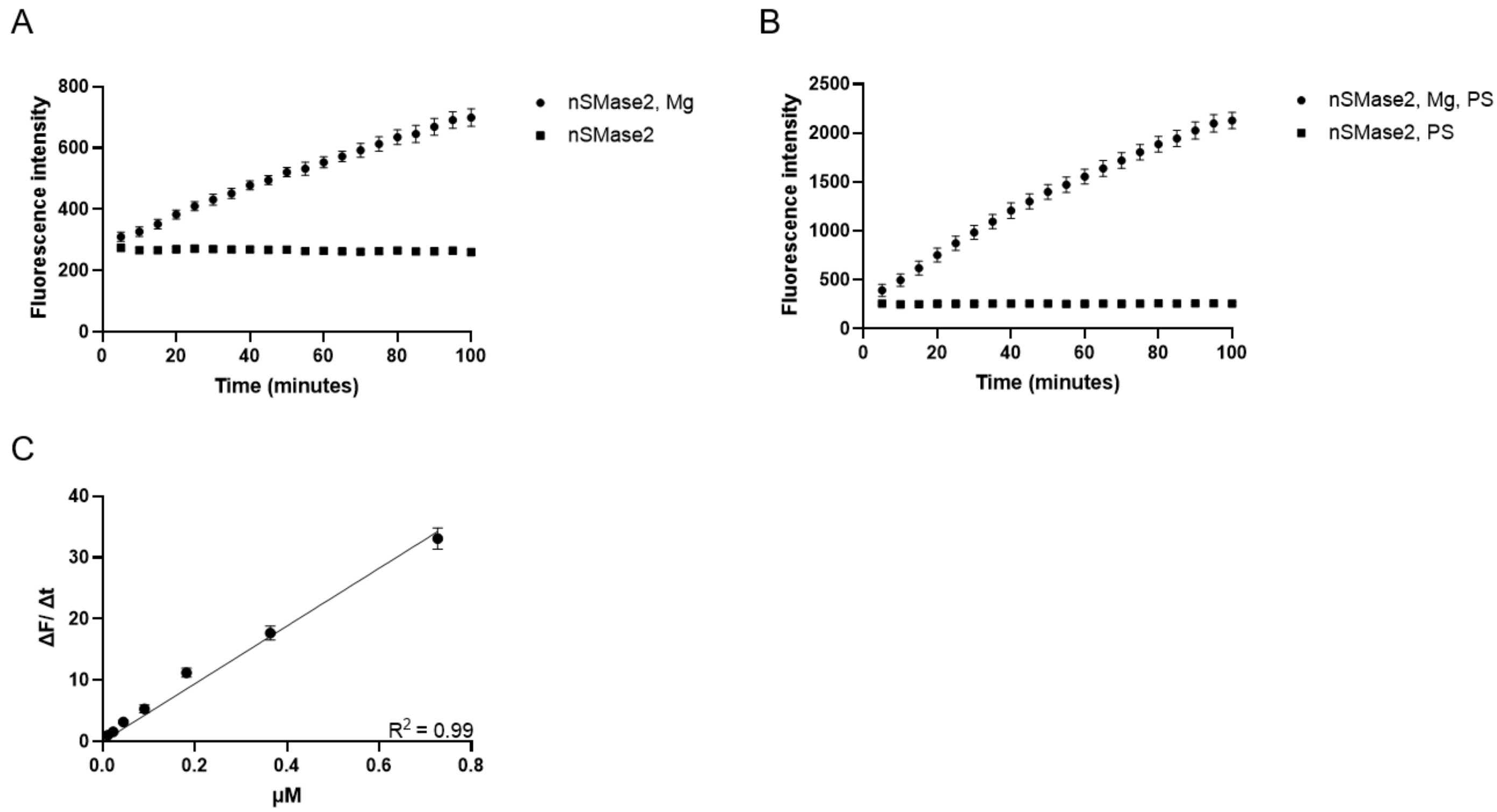


**Figure S2. Assay parameters time and enzyme linearity.** 0.182 μM recombinant nSMase2 ΔIns was incubated with 1 μM PCK, 5 mM $Mg^{2+}$ +/- 10 mol% PS. Negative control is WT nSMase2 without $Mg^{2+}$. Error bars indicate standard deviation from three independent experiments performed with technical duplicates (n=3). The reaction was linear with time up to 100 minutes without PS (A) and linear up to 50 minutes in the presence of PS (B). (C) Enzyme concentration linearity in the presence of 10 mol% PS.

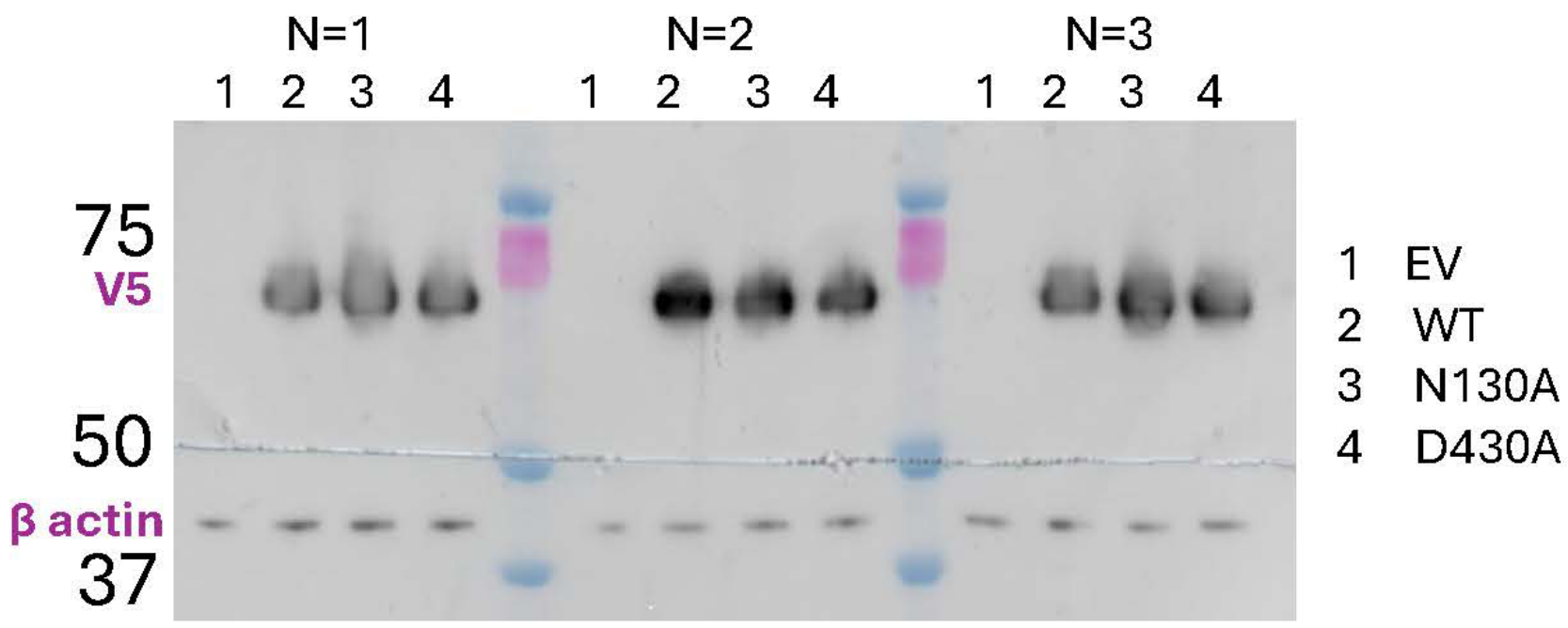


**Figure S3. Full, uncropped image of Western blot used for Figure 6A.** 1 = empty vector, 2 = wild type, 3 = N130A, 4 = D430A from 3 different transfection experiments.
